# The Impact of Tregs on the Anticancer Immunity and the Efficacy of Immune Checkpoint Inhibitor Therapies

**DOI:** 10.3389/fimmu.2021.625783

**Published:** 2021-02-26

**Authors:** Jose M. González-Navajas, Dengxia Denise Fan, Shuang Yang, Fengyuan Mandy Yang, Beatriz Lozano-Ruiz, Liya Shen, Jongdae Lee

**Affiliations:** ^1^Alicante Institute for Health and Biomedical Research (ISABIAL), Hospital General Universitario de Alicante, Alicante, Spain; ^2^Networked Biomedical Research Center for Hepatic and Digestive Diseases (CIBERehd), Institute of Health Carlos III, Madrid, Spain; ^3^Department of Pharmacology, Pediatrics and Organic Chemistry, University Miguel Hernández, Elche, Spain; ^4^Institute of Research, Development and Innovation in Healthcare Biotechnology in Elche (IDiBE), University Miguel Hernández, Elche, Spain; ^5^State Key Laboratory of Respiratory Disease, School of Basic Medical Sciences, Guangzhou Medical University, Guangzhou, China

**Keywords:** immune checkpoint inhibitor (ICI), Tregs, Treg-depletion, Foxp3, PD-1, CTLA-4

## Abstract

Although cancers arise from genetic mutations enabling cells to proliferate uncontrollably, they cannot thrive without failure of the anticancer immunity due in a large part to the tumor environment's influence on effector and regulatory T cells. The field of immune checkpoint inhibitor (ICI) therapy for cancer was born out of the fact that tumor environments paralyze the immune cells that are supposed to clear them by activating the immune checkpoint molecules such as PD-1. While various subsets of effector T cells work collaboratively to eliminate cancers, Tregs enriched in the tumor environment can suppress not only the native anticancer immunity but also diminish the efficacy of ICI therapies. Because of their essential role in suppressing autoimmunity, various attempts to specifically deplete tumor-associated Tregs are currently underway to boost the efficacy of ICI therapies without causing systemic autoimmune responses. A better understanding the roles of Tregs in the anti-cancer immunity and ICI therapies should provide more specific targets to deplete intratumoral Tregs. Here, we review the current understanding on how Tregs inhibit the anti-cancer immunity and ICI therapies as well as the advances in the targeted depletion of intratumoral Tregs.

## Introduction

ICI therapies targeting programmed death 1 (PD-1), programmed death ligand 1 (PD-L1), or cytotoxic T lymphocyte-associated antigen 4 (CTLA-4) have effectively treated various types of cancer. PD-1 is induced in activated T cells while its ligand PD-L1 is expressed in many cell types including antigen-presenting cells (APCs) and tumor cells. PD-1/PD-L1 interaction inhibits T cell survival and proliferation (1). CTLA-4 is also induced on activated T cells and has a much stronger affinity than CD28 to B7 molecules (CD80 and CD86) in APCs, thus competing with APCs for B7 and inhibiting full activation of T cells (2). Unfortunately, only a fraction of patients responds to these therapies, leaving the vast majority without benefit (3). While ICI therapies understandably have focused on reinvigorating cytotoxic responses by CD8^+^ or NK cells, growing evidence indicates that CD4^+^ T cell subsets have differential influences on the efficacy of ICIs (The multifaceted role of Th1, Th9 and Th17 cells in immune checkpoint inhibition therapy). One of the reasons for the poor response to ICIs in many patients has been attributed to intratumoral regulatory T cells (Tregs). As Tregs are essential in maintaining immune homeostasis and preventing autoimmunity, deficiencies in Treg development or function cause uncontrolled immune responses and autoimmune diseases (4). However, intratumoral Tregs may promote tumor progression by suppressing the natural anticancer immune responses. The percentage of Tregs among CD4^+^ cells is significantly higher in tumors compared to its share in the immune organs (5–8). The increased percentage of Tregs in many cancers is often associated with poor prognosis (9). Therefore, the impact of Tregs should be considered for successful ICI therapies. In this review, we discuss the role of Tregs in the anticancer immunity and ICI therapies as well as the strategies being developed to specifically deplete intratumoral Tregs.

## Brief Overview of Treg Development and Diversity

Tregs, originally identified by Sakaguchi et al. as CD4^+^CD25^+^ T cells essential for self-tolerance (10), are divided in several subsets in mice (Table 1). Natural regulatory T cells (nTregs) are induced by a broad spectrum of autoantigens in the thymus, thus also called tTregs. On the other hand, peripherally derived Tregs (pTregs) or induced Tregs (iTregs) are differentiated from naïve CD4^+^ T cells in the periphery or *in vitro*, respectively, by various factors including transforming growth factor-β (TGF-β), dendritic cells (DCs) expressing indole amine 2,3-dioxygenase (IDO), or retinoic acid (11). While the master transcription factor Foxp3 (forkhead box P3) expression in both subsets is essential for their suppressive function, there are important differences between them. For instance, tTregs express a high level of Helios and Neuropilin-1 (NRP1) whereas pTregs express very little of them (12, 13). There are two other CD4^+^ Treg subsets that do not express Foxp3, IL-10-producing T regulatory type 1 (Tr1) cells (14) and TGF-β-producing T helper type 3 (Th3) cells (15). In addition, there are CD8^+^ Tregs that express Foxp3, that are the first reported suppressor T cells, and regulatory B cells (Bregs) that suppress the proliferation of lymphocytes, including effector T cells mainly via secretion of IL-10. Herein, we will mostly focus on CD4^+^Foxp3^+^ Tregs. Treg heterogeneity was reviewed previously (16, 17). As non-Tregs cells in humans also express Foxp3 when acutely activated, Tregs are classified based on the expression of other markers such as CD45RA and CD127 in addition to Foxp3. Weakly suppressive naive Treg cells (nTreg: CD4^+^CD45RA^+^Foxp3^low^CD127^low^ cells) can differentiate into effector Tregs (eTreg: CD4^+^CD45RA^−^Foxp3^high^ cells), which are activated and highly suppressive (18, 19). In conclusion, Tregs come in a variety of different forms depending on species and locality (20), and thus are expected to express different sets of surface markers and functions, especially in different tumor microenvironments.

**Table 1:** Treg subsets.

**Species**	**Names**	**Markers**
Mouse	nTreg or tTreg	CD4^+^CD25^+^Foxp3^+^Helios^high^NRP1^+^
	pTreg	CD4^+^CD25^+^Foxp3^+^
	iTreg (*in vitro* generated)	CD4^+^CD25^+^Foxp3^+^
	Tr1	CD4^+^ Foxp3^−^IL-10^+^
Human	nTreg	CD4^+^CD45RA^+^Foxp3^low^CD127^low^
	eTreg	CD4^+^CD45RA^−^Foxp3^high^

*Foxp3, Forkhead box P3; Treg, regulatory T cell; nTreg (mouse), natural Treg; tTreg, thymus-derived Treg; pTreg, peripheral Treg; iTreg, induced Treg; Tr1, T regulatory Type 1; nTreg (human), naïve Treg; eTreg, effector Treg*.

## Suppressive Mechanisms of Tregs

Both nTregs and pTregs suppress the functions of T cells as well as other immune cells including B cells, NK cells, dendritic cells (DCs), and macrophages via humoral (IL-10, TGF-β, IL-35, granzyme B, adenosine, cAMP) and cell-cell contact mechanisms (CTLA4, GITR, LAG3). The detailed mechanisms were reviewed elsewhere (4). Although the local environment influences which of these mechanisms Tregs use to suppress immune responses, Foxp3-regulated genes are most likely essential for the Treg functions. Foxp3 controls the expression of IL-2, CD25 (IL-2 receptor α-chain), CD122 (IL- 2 receptor β-chain), and CTLA-4, and deficiency in any of them results in autoimmune diseases observed in Foxp3 deficiency (21). It is noteworthy that the canonical Th2 transcription factor GATA binding protein 3 (GATA-3) is important for maintaining Foxp3 expression and Treg suppressive function (22, 23).

Tregs rely on IL-2 produced by effector T cells for survival and proliferation since Foxp3 suppresses IL-2 expression (24). However, Tregs express a higher level of the high-affinity IL-2 receptor complex comprised of IL-2Rα (CD25), IL2Rβ (CD122), and IL-2Rγ (CD132) (10), which, acting as an IL-2 sink, can starve other T cells of IL-2. Therefore, exogenous IL-2 can rescue effector T cells from IL-2 depravation mediated by Tregs *in vitro* (25). Theoretically, IL-2 supplementation during ICI therapies may also relieve effector T cells of IL-2 depravation where Tregs are enriched. However, systemic administration of IL-2 at a high dose is risky as it can cause severe inflammation. A low dose IL-2 or a modified IL-2 that can only bind to the high-affinity IL-2 receptor can be considered to minimize the systemic effect (26–28), which can be useful for activating Tregs to suppress hyper-inflammation.

CTLA-4 is highly expressed in Tregs but also in activated effector T cells. CTLA-4 has a much higher affinity than CD28 for their common ligands CD80 and CD86, thus preventing CD28 in effectors T cells from being activated by CD80/CD86 in APCs (2). It also modulates T cells' motility and interactions with APCs (29). However, CTLA-4 regulates CD4^+^ and CD8^+^ cells differentially since Chambers et al. showed that lymphoproliferation in CTLA-4^−/−^ mice was due to costimulation-dependent activation of CD4^+^ cells (30). Ipilimumab (anti-CTLA-4 mAb) appears to be working in part by depleting Tregs via antibody-dependent cellular cytotoxicity (ADCC) in animal studies (31–33). Sharma et al. reported that anti-CTLA-4 Abs (Ipilimumab and Tremelimumab) did not deplete Foxp3^+^ cells within the tumor microenvironment, but suggested modifying the Fc region of the antibodies to enhance Fc-mediated depletion of intratumoral Tregs (34). Indeed, an anti–CTLA-4 mAb engineered for high ADCC depleted Treg cells more effectively (35). Although the clinical benefit of Ipilimumab treatment highly correlated with the decreased number of intratumoral Tregs (36), further studies are needed to determine the precise role of anti-CTLA-4 therapy has on intratumoral Tregs.

In conclusion, although Tregs use a host of different mechanisms to suppress T and other cell types, IL-2 depletion and up-regulation of CTLA-4 mediated by Foxp3 appear to be the most relevant in suppressing anticancer immunity.

## The Role of Tregs on Cancer Development

It is quite clear in mice that Tregs suppress tumor-specific effector T cells since mice depleted of Tregs by anti-CD25 antibody or T cell-deficient mice reconstituted only with effector T cells effectively eliminate a variety of syngeneic tumors (37, 38). Several lines of evidence suggest that this is also true in humans. Tregs are over-represented in a number of tumors compared to immune organs or blood (5–8). The reasons for the increased intratumoral Tregs numbers over effector T cells include recruitment via different chemokines secreted by various tumor cells and other cells in the tumor microenvironment (7, 39, 40), local expansion of Tregs activated by self-antigens presented by dying tumor cells (41, 42), the ability of Treg cells to adapt their metabolism to the tumor microenvironment (43, 44), and differentiation of naïve T cells to pTregs (45). Intratumoral Tregs are more powerful suppressors relative to Treg derived from the patient's autologous blood (46–48). The number of intratumoral Tregs is negatively correlated to poor prognosis (8). However, in cancers that share a common feature of prominent chronic inflammation, such as colon, breast, bladder, or head and neck cancers, accumulation of Tregs in tumors is associated with favorable prognosis by potentially suppressing tumor-promoting inflammation (7). The discrepancy may be attributed at least in part to the fact that non-Tregs also express Foxp3 when activated (9), and thus highly suppressive effector Tregs may have been over-estimated in some studies (8). Overall, Tregs suppress immune surveillance against tumor development and progression.

## Effects of Anticancer Immunotherapy on Tregs

The current immunotherapies target either PD-1/PDL-1 and/or CTLA-4 to energize effectors T cells. However, Tregs in the tumor microenvironment also express both PD-1 and CTLA-4 at a high level compared to effector T cells. PD-1 appears to inhibit the suppressive function of intratumoral Tregs. Indeed, PD-1 inhibition significantly promoted the proliferation of highly suppressive PD-1^+^ effector Treg cells in patients who developed hyperprogressive disease after the anti-PD-1 mAb Nivolumab treatment, resulting in inhibition of antitumor immunity (49). Initially it was thought that CTLA-4 blockade in Tregs would reactivate effector T cells to attack tumors by allowing CD28 in T cells to bind to B7 molecules in APCs. However, anti-CTLA4 antibody (Ipilimumab) preferentially depleted intratumoral Tregs by ADCC and antibody-dependent cellular phagocytosis (ADCP) (35). These findings suggested that a combination of PD-1 and CTLA-4 blockers is likely to synergize to activate intratumoral effector T cells by relieving effector T cells from PD-1/PDL-1-mediated anergy and depleting intratumoral Treg, respectively. Indeed, the combination of Ipilimumab (anti-CTLA-4) and Nivolumab (anti-PD-1) significantly enhanced efficacy in metastatic melanoma patients and probably is more effective in other cancers than monotherapies (50, 51). The combination therapy was approved for treatment of other cancers including metastatic melanoma, advanced renal cell carcinoma and metastatic colorectal cancer with MMR/MSI-H aberrations.

## Therapeutic Targets to Specifically Deplete Intratumoral Tregs

Several lines of evidence suggest that intratumoral Tregs are a major obstacle in ICI therapies. First, as mentioned above, the percentage of intratumoral Tregs among CD4^+^ T cells is higher than that of Tregs in peripheral blood; second, intratumoral Tregs show a highly activated suppressive phenotype (52); third, depletion of Tregs enhances anticancer immunity in mice and humans. Therefore, intensive efforts are ongoing to find a way to deplete intratumoral Tregs to enhance ICI therapies without activating autoimmune response. The targets should be either exclusively expressed or highly enriched in intratumoral Tregs, preferably on the surface.

CD25 (IL-2Rα) is constitutively expressed in Tregs and induced in activated effector T cells. Systemically targeting CD25 can cause severe inflammation, and thus alternative approaches to target CD25 exclusively in intratumoral Tregs are being investigated. For example, Vargas et al. found that upregulation of the inhibitory Fc gamma receptor (FcγR) IIb at the tumor site prevented intratumoral Tregs depletion by anti-CD25 antibodies. An anti-CD25 antibody with a higher affinity to activating FcγRs effectively depleted intratumoral Tregs cells and induced complete tumor rejection in combination with anti-PD-1 antibody (53). Sato et al. developed to a photoactivable anti-CD25 antibody that can be targeted by near-infrared radiation to deplete intratumoral Tregs (54). Recently, Solomon et al. reported efficient depletion of Tregs with an anti-CD25 antibody (RG6292) without inhibiting IL-2 signaling in effector T cells in both nonhuman primates and humanized mouse models, synergistically enhancing an ICI therapy but without overt immune responses (55).

Intratumoral Tregs express several surface molecules at a higher level than Tregs in normal tissues or blood such as PD-1, PD-L1, PD-L2, TIGIT, GITR, OX-40, TIM-3, and 4-1BB (56), suggesting they are activated and highly suppressive. Among them, activation of ICOS, 4-1BB, and GITR was shown to inhibit Treg suppressive function but stimulate effector T cells (57, 58), leading to several clinical trials. Agonistic anti-OX40 mAb (59) is being tried as a monotherapy or a combination therapy with ICI on solid tumors (NCT02221960). GITR agonists are also being tested on solid tumors alone or in combinations with ICIs (NCT02583165 and NCT02628574) (60). The agonistic anti-ICOS mAb JTX-2011 is currently being evaluated in a clinical trial (NCT02904226) alone or in combination with a fixed dose of Nivolumab in people with advanced solid tumors. It will be interesting to see if these therapies indeed work on tumors by inhibiting Tregs while activating effector T cells.

The fact that Tregs are enriched in the tumor tissues compared to other organs or blood led to search for chemokines and chemokine receptors essential to recruiting Tregs to tumors. Different tumors harbor Tregs expressing different chemokine receptors (7), therefore, the chemokine receptor expression pattern in intratumoral Tregs can be exploited to enhance ICI therapies. CCR4-expressing Tregs were shown to be attracted to CCL22 released by macrophages within ovarian cancer, which was associated with a decreased survival rate (39), and the anti-CCR4 mAb Mogamulizumab effectively depleted effector Tregs in humans to elicit anticancer response (61), which is now being trialed alone or in combination with ICIs in solid tumors (62). CCL28 induced by hypoxia in ovarian cancer recruited CCR10^+^ Tregs, promoting tumor tolerance and angiogenesis (40). In a murine model of pancreatic cancer, a blockade of the CCR5-CCL5 axis inhibited recruitment of Tregs and inhibited tumor growth (63). Specificity of Treg chemotaxis to tumors needs further investigation for clinical application.

Another approach to disable intratumoral Tregs is to selectively convert them into effector T cells, although this approach is still in an early stage of development. Several molecules are essential for maintenance of Treg functions besides Foxp3, including OX-40, GITR, the histone methyltransferase EZH2, and Helios. Tumor-infiltrating Tregs are dependent on EZH2, an epigenetic switch, to maintain Treg stability and function (64, 65). A small molecule inhibitor of EZH2 drove intratumoral Tregs to acquire pro-inflammatory functions, leading to remodeling the tumor microenvironment and enhancing the anticancer immunity without provoking systemic autoimmunity (66). Helios is essential for the maintenance of Treg stability under inflammatory conditions such as autoimmune diseases and cancers. CD4 Treg-specific deletion of Helios enhanced antitumor immunity by induction of an unstable phenotype and conversion of intratumoral Tregs into T effector cells within the tumor microenvironment (67).

Recently, Ho et al. found that CD36 was selectively upregulated in intratumoral Treg cells in lung cancers and melanomas and that CD36 deletion in Tregs suppressed tumor growth without causing systemic autoimmune response (68). In this study, as CD36 in Tregs was important to suppress the anticancer immunity, a monoclonal antibody that blocked CD36 from bonding to fatty acids and to oxidized LDLs induced apoptosis of Treg cells while promoting the accumulation of CD8^+^ T cells in the tumor. Whether this phenomenon is unique to certain cancers remains to be seen.

TGF-β is essential for development of Tregs as well as Th17 cells and was originally thought to be secreted primarily by cancer cells and/or Tregs in the tumor microenvironment. However, the major source of TGF-β in tumors was reported to be CD4^+^ Th cells (69–71). Recently Li et al. reported that TGF-β suppresses Th2 cell-mediated anticancer immunity in an autocrine fashion and that blocking TGF-β signaling in CD4^+^ T cells inhibited cancer progression (72). In this study, the effects of TGF-β blockade on Tregs were not analyzed. Identifying exclusive markers of intratumoral Th2 cells to deplete them or systemic TGF-β blockade can be considered in combination with ICIs.

In conclusion, a growing number of options to deplete or disable intratumoral Tregs are being developed and tested. Most of these approaches require a precise characterization of Treg phenotypes, which is not always possible. These approaches are summarized in Table 2.

**Table 2:** Approaches to deplete or disable intratumoral Tregs.

**Modes**	**Target**	**Drugs**
Checkpoint molecules	OX40	OX40 Agonist Ab MEDI0562 (59)
	ICOS	ICOS Agonist Ab JTX-2011 (73, 74)
	GITR	GITR Agonist Ab BMS-986156 (60)
	CTLA-4	Ipilimumab, ADCC-optimized anti-CTLA-4 Ab (35)
Blocking Treg chemotaxis to tumors	CCR4	Mogamulizmab (61)
	CCR5	Anti-CCR5 Ab (63)
	CCR10	Anti-CCR10 Ab immunotoxin (40)
Conversion of Tregs to effector T cells	EZH2	Several inhibitors including GSK343 (66, 75)
	Helios	An inhibitor of Helios to be developed
Others surface molecules	CD36	CD36 deletion Ab (68)
	CD25	Fc-optimized depletion CD25 Ab (53), photoactivable CD25 Ab (54)Treg-depleting CD25 Ab (RG6292) (55)

*Ab, Antibody; ADCC, antibody-dependent cellular cytotoxicity; CCR, C-C motif chemokine receptor; EZH2, Enhancer of zeste 2 polycomb repressive complex 2; CTLA-4, Cytotoxic T lymphocyte antigen 4; GITR, Glucocorticoid-induced TNFR-related protein; ICOS, Inducible T cell co-stimulator; OX-40, TNF receptor superfamily member 4*.

## Conclusion

There is a growing consensus that intratumoral Tregs promote tumorigenesis and progression and inhibit ICI therapies by suppressing anticancer immunity. The critical question is then how to disable or deplete Tregs inside tumors specifically to avoid systemic inflammation. Fortunately, there has been a significant progress in characterizing the phenotypes of intratumoral Tregs that are distinct from those of Tregs in the periphery or normal tissues. With continuing technical advances to take advantage of these findings to target intratumoral Tregs, the future is promising. However, some questions remain to be answered even if some of these approaches are eventually approved for clinical uses. Should Treg depletion be applied to all ICI therapies or selectively? What should be the selection criteria for the latter? Furthermore, all the cutting-edge medicines including ICI therapies are prohibitively expensive for the vast majority of patients, thus leaving most people in the developing countries out of reach. Therefore, more research should be done to bring down the cost of these medicines.

## Author Contributions

JL and JG-N conceived the manuscript idea and revised the manuscript content. BL-R, FY, LS, and DF edited the text and created the manuscript tables. All authors contributed significantly to the drafting and editing of this manuscript and read and agreed to the final version of the manuscript.

## Conflict of Interest

The authors declare that the research was conducted in the absence of any commercial or financial relationships that could be construed as a potential conflict of interest.
